# Hemophagocytic Lymphohistiocytosis Unmasking Systemic Lupus Erythematosus: Management With Belimumab and a Case Study

**DOI:** 10.7759/cureus.64596

**Published:** 2024-07-15

**Authors:** Anam Ahmad, Rama Atluri, Katherine J Robbins

**Affiliations:** 1 Internal Medicine, St. Luke's Hospital, Missouri, USA; 2 Rheumatology, St. Louis University, St. Louis, USA; 3 Pathology, St. Louis University, St. Louis, USA

**Keywords:** hyperferritinemia, belimumab, macrophage activation syndrome, systemic lupus erythematosus, hemophagocytic lymphohistiocytosis

## Abstract

Secondary hemophagocytic lymphohistiocytosis (HLH) is a life-threatening hyperinflammatory condition caused by the hyperactivation of macrophages and T-cells, triggered by infection, malignancy, or underlying rheumatological conditions. It rarely presents as a first manifestation of a rheumatological condition. Macrophage activation syndrome (MAS) is secondary HLH associated with underlying hematological conditions. Here, we present a case of a previously healthy 29-year-old female who was admitted with fever, rash, and pancytopenia, found to have HLH, and a workup revealed underlying systemic lupus erythematosus (SLE). She was successfully treated with dexamethasone, etoposide, and belimumab, with complete recovery of her symptoms. This case highlights the importance of a thorough evaluation of rheumatological conditions in all patients with HLH despite their previous medical history and the use of belimumab for SLE.

## Introduction

Hemophagocytic lymphohistiocytosis (HLH) is a hyperinflammatory condition, characterized by the proliferation of macrophage-like-histiocytes, and the inability of natural killer cells (NK) along with T lymphocytes to lyse the infected and antigen-presenting macrophages leading to a cytokine storm [1]. HLH can be familial or sporadic. Familial HLH is usually autosomal recessive due to a variation in the genes involved in lymphocyte granule-dependent cytotoxicity [2,3]. Rheumatological diseases, malignancies, or infections trigger secondary HLH. Secondary HLH triggered by rheumatological conditions is also known as macrophage activation syndrome (MAS). Among all the cases of HLH in SLE, the incidence of HLH ranges from 0.9-4.6% [4]. It is extremely uncommon to have HLH as the first presentation of SLE, and very few case reports have been described in the literature. We describe a case of a previously healthy 29-year-old female who presented to the hospital with HLH and was found to have SLE, a cascade of events that was triggered by infection.

## Case presentation

**Figure 1:**
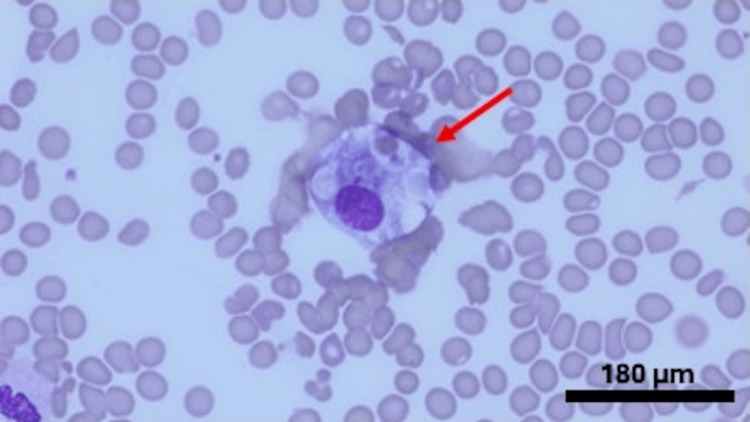
Bone marrow aspirate (Wright-Giemsa stain) x 1000; arrow revealing a histiocyte ingesting erythrocytes

**Figure 2:**
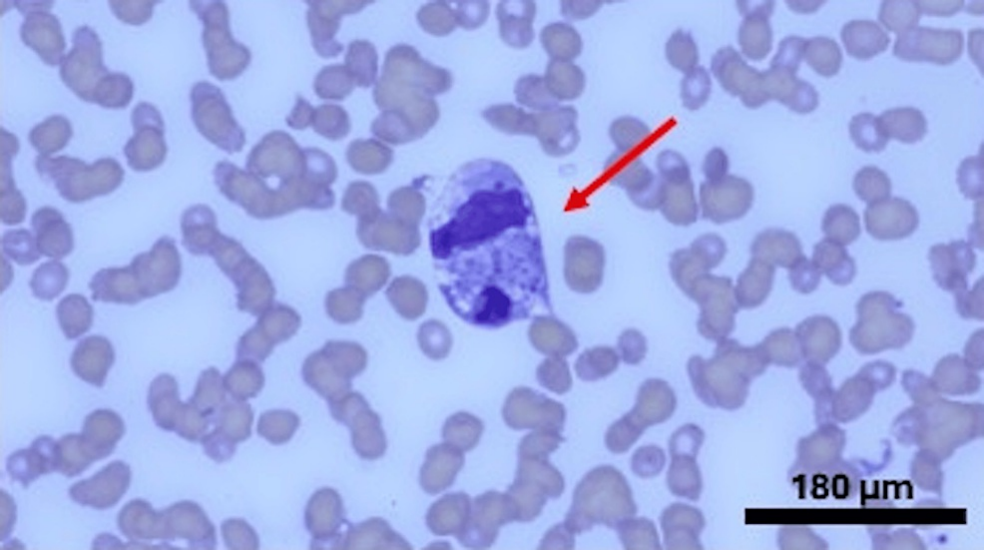
Bone marrow aspirate (Wright-Giemsa stain) x 1000, arrow revealing histiocyte with numerous platelets and a segmented neutrophil within its cytoplasm

A 29-year-old female with no significant past medical history was admitted to the hospital for a sore throat and rash on her face, followed by a fever with a maximum temperature of 103 °F from the last four to five days. She tried azithromycin prescribed by her primary care physician, which did not help. She mentioned malaise and loss of appetite but the rest of the review of symptoms was negative. Her family history was significant for SLE in her mother. Physical exam was significant for an erythematous and hyperpigmented maculopapular rash on the face and erythema on the hard palate. She was found to have pancytopenia, elevated inflammatory markers, including ferritin above 10,000 ng/ml, and transaminitis tests as described in Table 1. The patient had computed tomography of the chest and abdomen that revealed reactive prominent lymph nodes in the axilla, inguinal, and mesenteric regions. Her HScore revealed a probability of HLH of around 70-80% so she was started on dexamethasone 10 mg/m^2 ^daily. Meanwhile, the patient had a thorough infectious evaluation, including viral, bacterial, fungal, and atypical etiologies. She tested positive for Streptococcal A infection and Epstein-Barr virus. She also underwent an extensive rheumatological workup due to a significant family history, rash, and pancytopenia, which revealed underlying SLE. A biopsy of the rash also revealed vacuolar interface dermatitis. The biopsy of the bone marrow showed normocellular marrow identifying hemophagocytosis and ruling out any underlying malignancy, fulfilling another diagnostic criterion for HLH (Table 2 and Figures 1-2). She was diagnosed with hemophagocytic lymphohistiocytosis, presumably due to underlying SLE and triggered by the infections. She was treated with dexamethasone 10 mg/m2 daily and etoposide cycles along with hydroxychloroquine 200 mg daily, followed by the addition of belimumab 10 mg/kg every 4 weeks with marked improvement in fever, rash, cytopenia, and inflammatory markers.

**Table 1:** Laboratory Data

Laboratory test	Value	Reference Range
Hemoglobin	5.3	11.9-15.8g/dl
White Blood Cells (WBCs)	0.5	4.0-10.7 x 10E9/L
Absolute Lymphocyte Count (ALC)	0.07	1.00-4.00x 10E9/L
Absolute Neutrophil Count (ANC)	0.04	1.6-7.5 x 10E9/L
Platelets	74	150-429 x 10E9/L
Creatinine	1.23	0.56-0.96 mg/dl
Aspartate Aminotransferase (AST)	99	5-34 U/L
Alanine Aminotransferase (ALT)	64	5-55 U/L
Erythrocyte Sedimentation Rate (ESR)	113	0-20 mm/hr
C-Reactive Protein (CRP)	3.1	< 0.5 mg/dl
Ferritin	10,410	13-204 ng/ml
Fibrinogen	288	200-400mg/dl
Triglyceride	212	< 150 mg/dl
Lactate Dehydrogenase (LDH)	595	125-243 U/L
Direct Coomb’s Test (DAT)	Negative	Negative
Chemokine Ligand 9 (CXCL9)	5858	<647 pg/ml
Soluble Interleukin 2 Receptor (sIL2R)	735	223-710 u/ml
Urine Analysis	Neg for protein and red blood cells	Neg for protein and red blood cells
Urine Protein/Creatinine	<0.01	<0.01
Antineutrophil Antibody (ANA)	1:2560	<1:80
Anti-Double-Stranded Deoxyribonucleoprotein (dsDNA) Antibody	150	0-24 IU
Anti-Smith (Sm) Antibody	45	0-19 units
Anti-Chromatin Antibody	65	0-19 IU
Anti-Sjogren Syndrome-A (SSA) Antibody	160	<20 units
Anti-Sjogren Syndrome-B (SSB) Antibody	117	< 20 units
Complement 3	68	82-193 mg/dl
Complement 4	28	15-57 mg/dl
Rapid Strep A	Positive	Negative
Ebstein Barr Virus (EBV) Immunoglobulin (Ig) M	< 10	0.0-43.9 units/ml
EBV Polymerase Chain Reaction (PCR)	Detected < 500 units	Not detected
EBV IgG	>600	0.0-21.9 units/ml

**Table 2:** Proposed diagnostic criteria of HLH 2009 HLH: hemophagocytic lymphohistiocytosis

Molecular diagnosis of hemophagocytic lymphohistiocytosis (HLH)
Or diagnostic criteria of HLH (5/8 fulfilled)
1. Fever
2. Splenomegaly
3. Cytopenia (minimum 2 cell lines involvement)
4. Hemophagocytosis in bone marrow/ spleen or lymph node
5. Hyperferritinemia
6. Increased soluble interleukin 2 receptor
7. Absent or very decreased Natural Killer cells unction
8. Hypertriglyceridemia/ hypofibrinogenemia

Patients can also have hyponatremia or transaminitis (not included in the criteria)
(HScore takes into account these clinical and laboratory parameters to calculate a score that helps in determining the likelihood of HLH)

## Discussion

Secondary HLH due to rheumatological conditions, also known as macrophage activation syndrome, has been associated with systemic juvenile idiopathic arthritis and childhood-onset SLE in the pediatric population [3] and with adult-onset Stills disease in the adult population. SLE predisposes the patient to HLH later in the disease course usually [4]. It rarely presents as the first manifestation of SLE in the adult population. Rheumatological disorders trigger the inflammatory response by the formation of autoantibodies and immune complexes leading to overactivation of macrophages and T lymphocytes.

Patients with HLH are acutely ill with multiorgan involvement including persistent high-grade fever, rash, change in mental status, lymphadenopathy, hepatosplenomegaly, and hypotension [5]. Labs typically demonstrate cytopenia of at least two lineages, hyponatremia, elevated liver function tests, renal dysfunction, bleeding diathesis, hyperferritinemia, hypertriglyceridemia, and hypofibrogenemia [6]. Cytokines that are found at extremely high levels in HLH are chemokine ligand 9 (CXCL9), interleukins (ILs) 1, 6, 10, and 12, and soluble IL-2 receptors [7].

The HLH diagnosis can be challenging due to its overlapping features with sepsis and multiple organ dysfunction syndrome. Diagnosis is based on clinical presentation in the setting of elevated inflammatory markers [8], supported by evidence of hemophagocytosis within tissue. It is recommended to perform a bone marrow biopsy or other tissue biopsy to prove the presence of hemophagocytes, but sometimes, it is difficult to do in critically ill patients [9]. Our patient's bone marrow biopsy did reveal the presence of multiple histiocytes. HScore helps in calculating the probability of HLH weighting clinical and laboratory criteria (Table 2).

The treatment strategy is based on relieving symptoms, calming the cytokine storm, and treating the underlying cause, which requires a multidisciplinary approach. For primary HLH, the treatment is dexamethasone (starting from 10 mg/m^2^ daily), etoposide (starting from 150 mg /m^2^ twice weekly), cyclosporine (starting from 15 mg/kg), emapalumab (starting from 1 mg/kg every 3 days), and eventually hematopoietic stem cell transplantation [10,11]. For secondary HLH in the case of SLE, steroids, hydroxychloroquine (HCQ), along with monoclonal antibodies, such as rituximab or belimumab, can be used. Belimumab is an anti-B-lymphocyte-stimulator antibody, approved for the treatment of active, autoantibody-positive SLE. It is usually used as an add-on therapy along with the standard of care [12]. The dosage can be 200 mg/week subcutaneously or 10 mg/kg intravenously every two weeks for three doses followed by every four weeks; we used the latter one for our patient. Recently, data have also emerged for the use of the IL-1 inhibitor anakinra as a first-line agent, especially if given early in the disease course [13]. Our patient had a good response with dexamethasone and etoposide upfront followed by belimumab and HCQ for her underlying SLE.

HLH can be life-threatening if not treated properly, with a mortality rate ranging from 20-70% so treatment response should be monitored closely [14]. Reduction in fever, improvements in liver/kidney function and coagulation profile, improvement in myelosuppression, and down-trending ferritin and IL-2R can be good clinical indicators [15].

## Conclusions

Physicians should be aware that HLH/MAS can present as the initial manifestation of SLE in adult patients. When patients present with HLH/MAS and show concerning features suggesting an underlying connective tissue disease, they should undergo a thorough rheumatological evaluation, even in the presence of active infections, as both conditions can coexist. Belimumab can be considered for treatment alongside standard care in cases of active SLE presenting as HLH. Prompt recognition of the underlying etiology and aggressive upfront treatment is essential in managing this life-threatening condition effectively.
